# Association of PPAR**α** Intron 7 Polymorphism with Coronary Artery Disease: A Cross-Sectional Study

**DOI:** 10.5402/2011/816025

**Published:** 2011-04-07

**Authors:** Sreeja Purushothaman, V. K. Ajitkumar, R. Renuka Nair

**Affiliations:** ^1^Division of Cellular and Molecular Cardiology, Sree Chitra Tirunal Institute for Medical Sciences and Technology, Thiruvananthapuram 695 011, India; ^2^Department of Cardiology, Sree Chitra Tirunal Institute for Medical Sciences and Technology, Thiruvananthapuram 695 011, India

## Abstract

The allelic variants of peroxisome proliferator-activated receptor alpha (PPAR*α*) can influence the risk of coronary artery disease (CAD) by virtue of its effect on lipid metabolism. However, the role of PPAR*α* intronic polymorphism with CAD has received little attention. The association of allelic variants G/C at intron 7 of the PPAR-alpha gene with CAD was examined in a hospital-based Indian population. PPAR genotyping was performed in 110 male patients with CAD and 120 age and ethnically matched healthy males by PCR amplification of the gene followed by restriction digestion. Presence of C allele showed a positive association with CAD (OR = 2.9; 95% CI [1.65–4.145]; *P* = .009) and also with dyslipidaemia (OR = 2.95, 95% CI (1.5–4.39); *P* < .05). Impaired lipid metabolism in carriers of the PPAR*α* Intron 7C allele is possibly responsible for the predilection to CAD.

## 1. Introduction

The peroxisome proliferator-activated receptors (PPARs) are part of a superfamily of ligand-activated transcription factors involved in fatty acid oxidation and lipid metabolism. PPAR*α* is expressed in the liver, heart, skeletal muscle, and kidney and regulates lipid and lipoprotein metabolism. PPAR**α** is also involved in other biological processes, including the regulation of inflammatory and oxidative pathways [1].

The role of PPAR*α* in cardiovascular disease is an interesting area for research because of its association with fatty acid oxidation, lipid metabolism, and inflammation. In general, activation of the PPAR*α*, both by natural and synthetic ligands, is considered beneficial for cardiovascular health [1]. In an extensive review, Cresci [2] have suggested the association of PPAR-alpha allelic polymorphism with cardiovascular disorders. There is emerging evidence that variation in the gene encoding PPAR*α* contributes to interindividual variability in lipid levels and cardiac hypertrophy. Jamshidi et al. [3] demonstrated that PPAR*α* G/C polymorphism influences human left ventricular growth in response to exercise and hypertension. There is only one report on the association between the PPAR*α* G/C polymorphism and cardiovascular disease events. Flavell et al. [4] had investigated the association between G to C transversion in intron 7 of the PPAR*α* gene and progression of atherosclerosis in a Finnish population. They found that homozygotes of Intron 7C had increased risk of ischemic heart disease and C allele carriers had greater progression of atherosclerosis than did G allele homozygotes. This observation warrants further studies and a better understanding of the role of PPAR*α* gene polymorphism in cardiovascular disease. Therefore, this study was taken up with the objective of examining the association of PPAR-alpha Intron 7 G/C polymorphism (G2528C) with CAD in an Indian population.

## 2. Methods

### 2.1. Study Sample

 All consecutive male patients admitted to the cardiology ward of the Institute, with angiographically confirmed CAD, were enrolled in the study. Exclusion criteria were clinical evidence of congenital abnormalities, valvular disease, chronic renal failure, and malignancies. History of hypertension, diabetes, and dyslipidaemia was obtained from medical records. Subjects were defined as hypertensive if their systolic blood pressure was ≥140 mmHg, if their diastolic blood pressure was ≥90 mmHg, or if they were receiving any antihypertensive treatment [5]. Patients with fasting glucose ≥126 mg/dL or receiving antidiabetic drugs were considered diabetic. Dyslipidaemia was defined as plasma cholesterol concentration ≥200 mg/dL, triglyceride concentration ≥180 mg/dL, or intake of lipid lowering drugs. Demographic details such as smoking and history of heart diseases among parents and first-degree relatives were recorded by personal interview. Body weight and height were measured for calculation of Body Mass Index (BMI)—Body weight (Kg)/(Height (m))^2^. Unaffected age-matched healthy male relatives of the spouse without any of the cardiovascular risk factors formed the control for comparison. Relatives of the spouse were chosen because they were matched for ethnicity and socioeconomic background. Consent for inclusion in the study was obtained from the subjects. The research protocol was approved by the Ethics Committee of the Institute.

### 2.2. Genotyping

Two milliliters of blood was collected in a heparinised tube. Genomic DNA was extracted from the whole blood with the use of the Gene elute: Blood genomic DNA kit (Sigma). Genotyping was carried out by polymerase chain reaction (PCR) and restriction enzyme digestion. PPAR*α* genotyping was performed in 25 microlitre reaction mix containing 5 microlitre 5x GoTaq buffer, 200 micromole of each dNTP, 1.75 mmol/L MgCl_2_, 5 pmols of each primer, and 0.5 U GoTaq polymerase. PCR primers used by Jamshidi et al. [3] were adopted for the study. The primer sequences are Forward -ACAATCACTCCTTAAATATGGTGG and Reverse- AAGTAGGGACAGACAGGACCAGTA (Sigma). PCR amplification generated fragments of 266 bp. PCR products were digested with 3 U TaqI (Fermentas) for 16 hours at 65°C and resolved using 1.5% agarose gel electrophoresis to 216 bp and 50 bp in carriers of the C allele.

### 2.3. Statistics

Chi-square analysis was carried out to test whether the distribution of PPAR genotypes was in Hardy-Weinberg equilibrium. Odds ratio was calculated as a measure of association of the PPAR alleles with coronary artery disease. Pearson *χ* ^2^ test was used as the test of significance for the comparison of genotype and allele frequencies between patients and control. *P* < .05 was considered statistically significant.

## 3. Results

 The study sample included 110 patients and 120 controls. The mean age of the sample was 48.64 ± 11.04 years. As expected, blood pressure, BMI, and history of smoking and familial incidence of heart disease were higher in patients (Table 1). Among the patients, 31.8% were diabetic, 40.9% had hypertension, and dyslipidaemia was reported in 51.8%. All patients with dyslipidaemia were on lipid lowering drugs. None of the common risk factors were present in the control. A combination of the above risk factors was seen in 56% of patients. 

### 3.1. Association of PPAR*α* G/C Polymorphism with CAD

Genotype distribution was in Hardy-Weinberg equilibrium in the control sample (*χ* ^2^ = 0.14, *P* > .05) as well as among patients (*χ* ^2^ = 0.01, *P* > .05). The proportion of individuals with GC genotype was higher among patients compared to control (Table 2). There was only one CC homozygote in the patient sample; hence for statistical analysis it was pooled with GC. The positive association between GC genotype and coronary artery disease was highly significant. The presence of C allele appears to enhance the risk for development of CAD (Table 2). The frequency of C allele shows a threefold increase in patients compared to healthy control.

### 3.2. Association of PPAR*α* G/C Polymorphism with Risk Factors for CAD

Analysis of the relative risk associated with the presence of C allele in patients with and without the common risk factors for CAD showed that C allele significantly enhances the risk for dyslipidaemia (Table 3).

## 4. Discussion

Risk factors commonly associated with CAD were seen in patients (Table 1). A significant association was observed between coronary artery disease and PPAR*α* genotypes (Table 2). The risk for CAD was significantly enhanced in the presence of PPAR*α* C allele. The allele also showed a positive association with dyslipidaemia (Table 3). Peroxisome proliferator-activated receptor alpha (PPAR*α*) regulates genes involved in lipid metabolism, homeostasis and inflammation, in response to fatty acids, and fibrates, making it a candidate gene for risk of dyslipidaemia, atherosclerosis, and coronary artery disease. Various polymorphic markers that act as candidate genes are related to lipoprotein metabolism and accounts for variations in the level of total serum cholesterol and LDL [6–8]. Though the mutation (G→C) in Intron 7 of the PPAR*α* gene is in the noncoding region, studies have indicated its potential biological importance. Due to its location in the intron, the variant itself may not be functional, but it may be in allelic association with a functional variant in a promoter or enhancer element of the PPAR*α* gene that results in reduced PPAR*α* gene expression [3]. Intron 1C and intron 7C-alleles mark a haplotype with reduced PPAR*α* gene expression [9]. Earlier studies on the association of PPAR*α* Intron 7C polymorphism with dyslipidaemia in diabetic subjects have shown that variation in the PPAR*α* gene influences total cholesterol, LDL-cholesterol (LDL-C), and HDL-cholesterol (HDL-C) in diabetic patients [10, 11] and that the C allele is associated with increased total cholesterol and LDL cholesterol [12]. Fibrates are used in the treatment of dyslipidaemia and cause a reduction in plasma triglycerides. Clinical trials have shown that fibrates reduce the progression of atherosclerosis [13] and the incidence of CAD [14, 15]. Reduced response to fibrate therapy has been reported in individuals with the C allele [11]. Genetically determined reduced PPAR*α* level associated with the presence of C allele may be the determining factor for impaired lipid metabolism, which in turn enhances the risk of CAD.

## 5. Conclusion

The study has demonstrated that PPAR*α* allelic variants influence the predisposition for CAD in human subjects, probably mediated by modulation of the lipid profile. Variations in the human PPAR*α* gene may be one of the contributory factors in the multifactorial etiology of CAD with carriers of the C allele having a predilection to the disease due to raised serum cholesterol. More extensive epidemiological and genetic studies are needed to understand the relationship between the G/C polymorphism in the PPAR*α* gene and serum lipid levels. It is also envisaged that studies will be carried out in other populations to establish the power of the association and also understand the cause for the reduced response of C allele carriers to fibrate therapy.

## Figures and Tables

**Table 1:** Demographic variables in patients with Coronary artery disease (CAD) and healthy control.

	Control	CAD
Sample size	120	110
SBP (mean ± SD)	126.02 ± 7.83	152.16 ± 8.86*
DBP (mean ± SD)	80.50 ± 5.25	98.72 ± 5.04*
BMI	23.69 ± 2.80	27.01 ± 1.78*
Smokers (%)	34	55*
Consanguinity (%)	5	8
Family history of heart disease (%)	19	29*

*P < .01 compared to control.

**Table 2:** Distribution of PPAR*α* intron 7C variants in patients with coronary artery disease (CAD).

	*n*	Genotype		Allele frequency	
		GC (%)	OR (95% CI)	C	OR (95% CI)
Control	120	6.67		0.03	
CAD	110	17.27*	2.92 (1.62–4.22)	0.09**	2.9 (1.65–4.145)

*P = .012,  **P = .009 versus control.

**Table 3:** Frequency of C allele in coronary artery disease patients with known risk factors.

Risk factors	*n*	C Allele frequency	Odds Ratio (95% CI)
Hypertension	45	0.11	1.5 (0.796 –2.2)
Diabetes	35	0.13	2.28 (1.15–3.41)
Dyslipidaemia	57	0.15*	2.95 (1.5–4.39)

*P < .05 versus patients with normal lipid profile.
